# The effect of *Vachellia eriolaba* leaf meal inclusion on growth performance, blood parameters and methane gas emission in lambs fed diets containing ammoniated maize stover

**DOI:** 10.1007/s11250-024-04149-x

**Published:** 2024-10-03

**Authors:** G. M. Chelopo, U. Marume

**Affiliations:** 1 School of Agricultural Science, Faculty of Natural Sciences and Agriculture, North-West University, P. Bag X 2046, Mmabatho, 2735 South Africa; 2 Food Security and Safety Niche area, Faculty of Agriculture, Science and Technology, North-West University, P Bag X 2046, Mmabatho, 2735 South Africa

**Keywords:** Lambs, Growth performance, Haematology, Methane gas emission, Serum biochemistry

## Abstract

The study evaluated the effect of *Vachellia erioloba* leaf meal in diets containing ammoniated maize stove on growth performance, methane emission and heath of growing lambs. Thirty-two female lambs were allocated to the following four dietary treatments: total mixed ration (TMR, control), 20% inclusion of untreated maize stover (UMS), 20% inclusion of ammoniated maize stover (AMS), and combined inclusion of 10% ammoniated maize stover and 10% *Vachellia erioloba* leaves (AMS*VL*). Each treatment was replicated 8 times and a lamb in an individual pen was regarded as an experimental unit in a completely randomized design. Feed intake was higher (*P* < 0.05) in lambs fed the AMS and AMSVL diets compared to those fed UMS. Final body weights were higher in lambs fed the AMS and AMSVL diets. Both average daily gain (ADG) and feed convention ratio (FCR) were not affected by diet. In comparison with the AMS and AMSVL diets, the lambs fed the UMS diet had the highest (*P* < 0.05) methane emission. Overall, lambs fed the control diets had the lowest (*P* < 0.05) methane gas emission. Blood hematological values were affected by diet with the AMSVL fed lambs having the highest (*P* < 0.05) mean platelet volume (MPV) and procalcitonin (PCT) values. Furthermore, total albumin, amylase and total bilirubin were the highest (*P* < 0.05) in lambs fed on the AMSVL diet. Lambs fed on AMS diet had the highest (*P* < 0.05) serum urea levels. It can be concluded that combined inclusion of ammoniated maize stover and *Vachellia* leaves improved feed value and lamb performance when compared to the individual inclusion of both UMS and AMS.

## Introduction

Maize stover is the major crop residue that can significantly contribute to the feed resource base for livestock. However, its utilisation by ruminants is often limited by high levels of lignified matter that reduce its digestibility (Lynch et al. 2014). Coupled with reduced digestibility, its utilisation may result in the production of high amounts of absolute methane (CH_4_) emission that contribute towards global warming (Aluwong et al. 2011). According to Aluwong et al. (2011) and Eckard et al. (2010) ruminants fed on grass or crop residues produce more CH_4_ than ruminants fed on legumes forage. Energy loss through CH_4_ production represents a significant loss of dietary energy that could potentially be redirected towards the meat production (Eckard et al. 2010). Globally it has been estimated that ruminants produce 80 million tons of CH_4_ annually, which accounts for 28% of anthropogenic emissions (Beauchemin et al. 2008; Metawi et al. 2017). This accounts for about 6–10% of the gross available energy from feed consumed by the animal.

Previous studies have shown that physical, biological and chemical treatment improve the chemical composition and digestibility of the stover (Arora et al. 2011; Buthane et al. 2021; Chelopo and Marume 2022; Wan Zahari et al. 2003). The ammoniation of crop residues is one of the chemical strategies to improve utilisations of poor-quality residues, by increasing the non-protein nitrogen (NPN) (Huang et al. 2022; Kraiem et al. 1991), that in turn will improve the reticulo-ruminal microbial activity. Furthermore, ammoniation can suppression mould growth, inhibition of bacterial proliferation of the stover during storage therefore improving the quality of the feed (Belanche et al. 2021; Huang et al. 2022; Spanghero et al. 2017). Ammoniation has been observed to increase the protein content whilst breaking down the cell wall matrices of the stover, ultimately decreasing the neutral detergent fiber (NDF) content, hemicellulose fraction and subsequent removal from cell wall constituents (Abera et al. 2018; Chelopo and Marume 2022). Nevertheless, for better utilization of ammoniated roughages, the inclusion of protein rich - fodder tree leaves can beneficial in complementing the stover (Abera et al. 2018; Liu et al. 2001; Marume et al. 2012; Mnisi and Mlambo 2017). The use of fodder trees such as *Vachellia sp* which are abundantly available during dry seasons in animal feeding systems has the potential to ameliorate problems of feed shortages for ruminants (Mlambo et al. 2008). Natural fodder trees such as *Vachellia erioloba* require limited agronomic inputs to grow and they are rich in readily fermentable nitrogen and energy (Kabir et al. 2004; Ravahuhali et al. 2020). Besides improving nutritive values of maize stover, the inclusion of leaf meal may help reduce CH_4_ gas emission. Methane gas emission from ruminants is a global concern. According to Ningrat et al. (2018) tannins are naturally occurring compounds that can act as anti-methanogenic agents in the rumen, therefore, could reduce CH_4_ production by ruminants. It is therefore hypothesized that the inclusion of *Vachellia erioloba* leaf meal in diets improves nutritive value and performance in lambs and reduces CH_4_ gas emission in lambs. Therefore, this study aimed at examining the effects of *Vachellia erioloba* foliage leaves inclusion in diets on the growth performance, blood parameters and CH_4_ gas emission in lambs fed ammoniated maize stover based diets.

## Materials and methods

### Study site and ethical considerations

The study was conducted at North-West University (NWU) Mafikeng Campus, South Africa. The North West Province is situated between 25° and 28° south latitude and between 22° and 28° east longitude, 1500 m above sea level.

### Ammoniation process

Two tons of stover obtained from the local commercial farms, were placed in airtight polyvinyl chloride plastic bags using garden forks. and subsequently tightly closed. Ammonia gas was then introduced through a pipe that pressed through the polyvinyl chloride into the middle of the sack, at a rate of 3.5% of the total dry matter. The opening left in the polyvinyl chloride bags after the withdrawal of the pipe was sealed with tape. The treated material was incubated for 4 weeks, in accordance with ammonia treatment procedures by Sundstoel et al. (1978). At the end of incubation period, samples of treated maize stover together with the untreated stover were obtained for the analysis of dry matter (DM), organic matter (OM), neutral detergent fibre (NDF), acid detergent fibre (ADF), and total nitrogen (N) at the North-West University Farm (Molelwane) in the Animal Science Research laboratory. The chemical composition of untreated maize stover (UMS) and ammoniated maize stover (AMS) is presented in Table 1.

**Table 1:** Chemical composition (%) of maize stover (MS), ammoniated maize stover (AMS) and *Vachellia erioloba*

Parameter	MS	AMS	Vachellia erioloba
DM	60.0	58.0	95.0
CP	3.59	9.23	13.0
EE	1.45	1.72	-
NDF	79.1	71.5	55.0
ADF	56.8	45.3	41.3
Total phenols	-	-	18.1
Non-tannin phenols	-	-	3.9
Total tannin	-	-	14.8
Condensed tannin	-	-	6.35
Hydrolysable tannin	-	-	8.49

DM = dry matter, CP = crude protein, EE = Effective energy, NDF = neutral detergent fibre ADF = acid detergent fibre

### V. Erioloba leaf harvesting and processing

*Vachellia erioloba* leaves were collected at the North-West University farm, Molelwane. The leaves were collected at an advanced maturity stage during the rainy season. The branches were clipped from the growing point; placed on polyethylene sheets and air-dried for 3 days by placement under a shade to prevent nutrient heat damage of heat-sensitive nutrients and turned regularly to prevent a build-up of moulds. The leaf meal was obtained by carefully shaking the branches and passed through a 2 mm sieve to eliminate thorns and pods and subsequently milled. Milled leaf samples of *Vachellia erioloba* species were analysed for DM, OM, NDF, ADF, and N at the North-West University Farm (Molelwane) in the Animal Science Research laboratory. The chemical composition of harvested *Vachellia erioloba* leaf is represented in Table 1. Total phenols and total tannins concentrations in the extract were determined by a modification of the Folin-Ciocalteu method (Makkar 2003) using polyvinylpolypyrrolidone (PVPP) to separate tannin phenols from non-tannin phenols, and condensed tannins (CT) were determined by the butanol-HCl-iron method. Both total phenols and total tannins were expressed as tannic acid equivalent and condensed tannins as leucocyanidin equivalent. The hydrolysable tannin was calculated as the difference between total tannin and CT.

### Dietary treatments

Four isnonitrogenous and isoenergetice diets were formulated to meet the requirements for growing lambs with the inclusion of ammoniated maize stover and *A. erioloba* leaf meal. A conventional lamb finisher diet (total mixed ration, TMR) was used as the control diet (Control). The fibre and protein component of the total mixed ration was replaced with untreated maize stover (UMS), ammonia-treated maize stover (AMS) and a combination ammonia-treated maize and *Vachellia* leaves (AMSVL) as follow:

- TMR: Conventional lamb finisher diet (TMR) (Control).
- UMS: TMR + 20% untreated maize stover.
- AMS: TMR + 20% treated maize stover.
- AMSVL: TMR + 10% treated maize stover: + 10% *Vachellia* leaves.

The dietary formulation and chemical compositions are shown in Table 2.

**Table 2:** Proportion of ingredients and chemical composition of the experimental diets

Ingredients (%)	Control	UMS	AMS	AMSVL
Lucerne	10	0	0	0
Hay	10	0	0	0
Maize stover	0	20	0	0
Ammoniated maize stover	0	0	20	10
*Vachellia* erioloba leaves	0	0	0	10
Maize/Chop	52.3	49.1	45.2	51
Wheat bran	10	10	15	12
Molasses meal	10	10	10	8.6
Soybean OCM	5	8	7	6
Feed grade urea	1	1.3	1.1	1.2
Feed limestone	0.7	1.1	1.2	0.7
Vitamin/mineral premix	1	1	1	1
ME MJ/kg	11.7	11.5	11.4	11.7
CP	15.3	15.1	15.4	15.6
CF	9.07	9.95	11.3	9.56

Experimental diets: Diet 1 (Control, lamb finishing ration, LFR), Diet 2 (20% maize stover, UMS), Diet 3 (20% ammoniated maize stover, AMS) and Diet 4 (10% ammoniated maize stove: 10% *Vachellia* leaves, AMSVL). ME: metabolizable energy, CP: crude protein, CF: crude fibre. The diets were formulated to be iso-caloric and iso-nitrogenous, using Format^®^

### Animal management and experimental design

Thirty-two female meatmaster lambs (aged 5–6 months) were used in this study. The lambs were randomly assigned to four dietary treatments in a complete randomized design. Each treatment had 8 replications with the lambs housed individually as experimental units. Feed and water were provided *ad libitum* throughout the experimental period. Seven days for acclimatization was allowed for the lambs to adapt to the diets and the study ran over 63 days.

### Feed intake and growth performance measurements

Feed intake was measured daily by subtracting the weight of feed leftovers from that of the feed offered, and thereafter, average daily feed intake (ADFI) was calculated as follows.

$$ADFI\, = \,\frac{{{\bf{\it{feed}}}\,{\bf{\it{offered}}} - {\bf{\it{feed}}}\,{\bf{\it{refusals}}}}}{{{\bf{\it{No}}}.{\text{\,}}\,{\bf{\it{of}}}\,{\bf{\it{days}}}}}$$

The initial weight of the lambs was recorded at the start of the study. Thereafter weekly weights were measured in the morning before feeding at 08H00 for two months. The average daily weight gain (ADWG) was calculated as:

$$ADWG\, = \,\frac{{{\bf{\it{Finish}}}\,{\bf{\it{weight\,}}}\left( {{\bf{\it{kg}}}} \right)\, - \,{\bf{\it{Start}}}\,{\bf{\it{weight}}}\left( {{\bf{\it{kg}}}} \right){\bf{\it{\,}}}}}{{{\bf{\it{No}}}.\,{\bf{\it{of}}}\,{\bf{\it{days\,}}}}}$$

Where: t0 = initial time, T = final time, W (T) = final body weight, and W (t0) = initial body weight.

Feed conversion ratio (FCR) per animal was also determined using the following formula:

$$FCR\, = \,\frac{{Feed\,\,intake\,\,\left( {kg} \right)}}{{weight\,\,gain\,\,\left( {kg} \right)}}$$

### Methane emission measurement

The CH_4_ gas production was measured on the lambs using a portable intelligent gas detector, pointing the red laser beam at one nostril of the lamb (IGD) (Tokyo Gas Engineering Co., Ltd. Anritsu Devices Co., Ltd., Tokyo, Japan) in ppm/ minute (Zhao et al. 2020; Sorg 2022). Measurements were repeated three times (during morning after feeding, midday and in the afternoon to account for any potential effect on methane emissions due to rumen fill. The measurements were taken bi-weekly throughout the experimental period.

### Blood hematological analyses

Blood samples were collected a day before experimental termination from the jugular vein using bleeding needles in a purple top vacutainer tube containing Ethylenediaminetetraacetic acid (EDTA) as an anticoagulant a day before the termination of the feeding trial. Blood was analyzed for hematology (erythrocyte count, hematocrit, hemoglobin, neutrophils, lymphocytes, monocytes, eosinophils, reticulocytes, white blood cells (WBC), and basophils) using an automated IDEXX LaserCyte Hematology Analyzer.

### Serum biochemical analysis

About 8 mL of whole blood samples were collected a day before experimental termination in red top vacutainer tubes, (vacuette^®^ tube, 8 ml, cat serum clot activator, 13 × 75, red cap-black ring, non-ridged, Lasec, Cape town, SA) that allow for clotting. The blood samples were then centrifuged at 2000 × *g* for 10 min at 15 °C within 4 h after collection to obtain serum, which was immediately stored in Eppendorf tubes in multiple aliquots at − 20 °C until analysis. The serum samples were analysed for enzyme activity, Calcium (Ca), cholesterol (Chol), creatinine (Crea), globulin (Glob), Glucose (Glu), Lipase (Lipa), Phosphorus (Phos), Total Bilirubin (Tbil), total protein (TP) and serum urea nitrogen (SUN), were analysed using an automated IDEXX Vet Test Chemistry Analyser (IDEXX Laboratories, Inc.).

### Statistical analysis

The general linear model’s procedure (PROC GLM) of SAS (2012) was used to analyze the effects of diet on growth performance, CH_4_ emission and blood parameters while the mixed model’s procedure for repeated measures of SAS (2012 was used to analyze the effect of diet on cumulating weight gain considering the effect of a week of measurement and diet. The following models were used:

$${\text{Yij}}\, = \,\mu \, + \,{D_i}\, + \,{\varepsilon _{ij}}$$

Where Yij = individual observation of the *i*-th Diet, µ = overall mean, D_i_ = effect of diet, εij = random error.

$${Y_{ijk}}\, = \,\mu \, + \,{D_i}\, + \,{W_j}\, + \,{\left( {D\,*\,W} \right)_{ij}}\, + \,{\varepsilon _{ijk}}$$

Where: *Y*_*ijk*_ = observation (Cumulative weight gain parameter), µ = population mean constant common to the observation, *D*_*i*_ = effect of diet, *Wj =* effect of week of measurement, D*W_*ij*_ = effect of diet interacting with week and ε_*ij*_ = random error term. For all tests, the level of significance was set at (*P* < 0.05).

## Results

### Growth performance measures and Ch4 emission of lambs

**Fig. 1:**
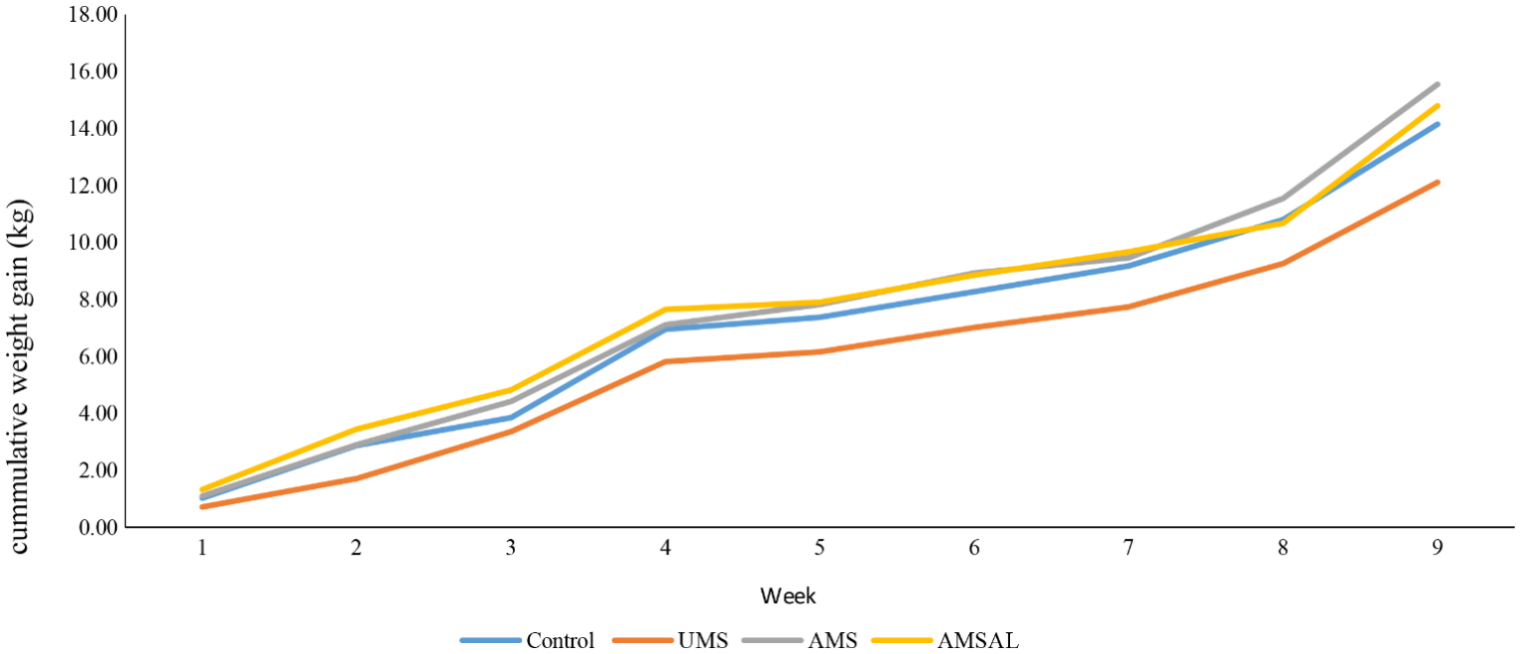
Effect of inclusion of ammoniated maize stover and *Vachellia* leaves in diets on cumulative weight gain of lambs

The effects of diet treatment on growth performance measures and CH_4_ emission of lambs are presented in Table 3. The results showed that while there were no differences in initial weights, the lambs fed the AMS and AMSVL diets had higher (*P* < 0.05) final body weights. Feed intake was, however, highest (*P* < 0.05) in lambs fed the control diet followed by those fed on AMSVL (*P* < 0.05) them those on AMS diet. The lambs fed untreated maize stover had the lowest (*P* < 0.05) feed intake compared to all other diets. Inclusion of *AL* had a considerable impact on feed intake. Feed intake in AMSVL fed lambs was significantly higher than that of the AMS fed lambs. No effect of diet was observed on ADG and FCR although the values for ADG were slightly higher in lambs fed the AMS and AMSVL diets while having slight slower FCR. Lambs fed a diet with UMS had the highest (*P* < 0.05) Ch_4_ gas emissions while the lowest CH_4_ gas emission was observed in lambs fed the control diet. Generally, there was a decline (*P* < 0.05) in CH_4_ gas emission with the inclusion of treated maize stover and the leaf meal. The lambs fed the AMSVL diet had lower (*P* < 0.05) CH_4_ gas emissions in comparison with those fed the AMS diet. In all the dietary treatments, cumulative weight gain (CWG) increased linearly from week 1 to week 9 (Fig. 1). The lambs fed the UMS diet had the lowest CWG compared to all the other treatments throughout the trial. Lambs fed AMSVL appeared to have a similar growth trend to that of those fed the control. The final weight was generally high in lambs fed on AMS followed by those fed the AMSVL diet with the lambs fed the UMS diet having the lowest end weight.

**Table 3:** Growth performance and methane emission of lambs’ fed on the inclusion of untreated maize stover, ammoniated Maize and inclusion of ammoniated maize stover and *Vachellia* leaves in a complete ration

Parameters	Control	UMS	AMS	AMSVL	SEM	*P*-value
Initial body weight (kg)	20.55	22.79	21.95	22.13	3.514	0.58
Final body weight (kg)	34.08	35.34	37.50	36.93	3.421	0.40
ADFI (kg)	1.39^a^	1.21^d^	1.27^c^	1.32^b^	0.043	0.0002
ADG (kg)	0.22	0.20	0.24	0.24	0.046	0.36
FCR (kg/kg)	6.54	6.41	5.36	5.94	1.291	0.53
Methane emission (CH_4_) (ppm)	75.38^c^	200.57^a^	148.13^b^	126.88^b^	32.740	0.0001

^a, b^ Means in the same row with different superscripts differ (*P* < 0.05) are significantly different. ADFI = Average daily feed intake, ADG = Average daily gain, FCR = Feed conversion ratio. ^2^Experimental diets: AMS0 (control), UMS (20% untreated maize stover), AMS (20% ammoniated maize stover) and AMSVL (10% ammoniated maize stover: 10% *Vachellia* leaves)

### Hematological parameters

The results on hematological parameters are presented in Table 4. Hematological blood parameters were affected (*P* < 0.05) by the inclusion of AMSVL in diets. Red blood cell count (RBC), hematocrit value and basophil granulocytes (BASO) were lower (*P* < 0.05) in lambs fed the AMSVL diet. Nevertheless, AMSVL fed lambs had higher (*P* < 0.05) mean platelet volume (MVP) and procalcitonin (PCT) compared all other lambs. No significant differences were observed in other hematological parameters across treatments.

**Table 4:** Effect of feeding experimental rations on lambs’ hematological parameters

Parameters	Control	UMS	AMS	AMSVL	SEM	*P*-value
Red blood cell (X10^12/L)	10.69^a^	10.82^a^	12.39^a^	8.57^b^	3.416	0.19
Hematocrit value (%)	19.85^ab^	19.75^ab^	23.49^a^	16.49^b^	6.329	0.20
Hemoglobin concentration (g/dL)	12.15	11.80	11.91	11.64	0.950	0.71
Mean corpuscular volume (fL)	18.55	18.28	19.04	19.41	1.128	0.37
Mean corpuscular hemoglobin (pg)	13.76	11.25	10.70	15.83	5.531	0.24
Red cell distribution width (%)	22.79	22.09	22.09	22.46	2.262	0.93
White blood cells (x10^9/L)	6.79	7.45	6.17	7.56	2.040	0.51
Neutrophil granulocyte (%)	28.96	33.06	32.31	31.16	8.965	0.82
Lymphocyte (%)	58.76	54.21	54.30	56.51	9.569	0.76
Monocytes (%)	6.85	6.48	6.75	7.59	1.674	0.72
Eosinophil granulocytes (%)	7.33	5.43	5.60	3.99	4.121	0.53
Basophil granulocyte (%)	0.93^ab^	0.89^ab^	1.06^a^	0.79^b^	0.241	0.17
Platelets (k/µL)	289.88	240.75	258.00	310.13	8.490	0.84
Mean platelet volume (fL)	6.54^b^	5.96^b^	5.96^b^	9.04^a^	2.411	0.01
Platelet distribution width (%)	23.96	24.53	23.06	22.28	4.324	0.73
Procalcitonin (%)	0.18^ab^	0.13^b^	0.15^b^	0.25^a^	0.088	0.05

^ab^ Means in the same row with different superscripts (*P* < 0.05) are significantly different. SEM: Standard error of the mean. Experimental diets: Control (TMR), UMS (20% untreated maize stover), AMS (20% ammoniated maize stover) and AMSVL (10% ammoniated maize stover: 10 *Vachellia* leaves)

### Serum biochemical profile

The results on serum biochemical parameters are presented in Table 5. Diet had no effect on liver enzyme activity (ALT and ALP) irrespective of treatments. However, amylase activity was observed to be higher (*P* < 0.05) in lambs fed on AMSVL as compared to those fed the control diet. Total bilirubin (TBIL) was higher (*P* < 0.05) in lambs fed AMSVL diet while urea concentration was highest in the lambs fed the AMS diet. The lambs fed the control diet had the lowest values for TBIL and urea concentrations. The level of serum glucose, phosphorus, cholesterol, creatinine, calcium and total protein were similar across the four diets. Total albumin was also no affected by diet although the values were observed to be lower in lambs fed on UMS diet.

**Table 5:** Effect of feeding experimental rations on lambs’ serum biochemical parameters

Parameters	Control	UMS	AMS	AMSVL	SEM	*P* – value
Albumin (g/L)	26.13^ab^	24.00^b^	26.29^ab^	28.25^a^	2.980	0.05
Alkaline phosphatase (U/L)	297.50	292.63	297.43	319.25	97.271	0.95
Alanine transaminase (U/L)	19.88	24.63	21.43	21.88	12.211	0.89
Amylase (U/L)	16.75^b^	20.98^ab^	22.43^ab^	25.50^a^	5.712	0.04
Lipase (U/L)	179.75	170.50	140.00	175.63	63.410	0.63
Calcium (mmol/L)	2.04	1.70	1.81	1.93	0.505	0.58
Cholesterol (mmol/L)	1.20	1.77	0.92	0.92	9.520	0.41
Urea (mmol/L)	7.90^bc^	9.51^ab^	11.20^a^	6.83^c^	1.230	0.03
Glucose (mmol/L)	2.53	2.43	2.53	2.43	0.554	0.98
Phosphate (mmol/L)	3.589	4.21	3.81	3.59	10.666	0.54
Total bilirabin (µmol/L)	2.75^b^	3.10^b^	3.57^b^	6.25^a^	2.336	0.02
Creanine (µmol/L)	49.50	51.50	59.00	52.00	9.989	0.31
Total Protein (g/L)	68.25	56.63	65.86	68.63	13.306	0.26
Globulin (g/L)	48.00	45.43	45.43	51.75	11.398	0.68

^ab^Means with different superscript letters within treatments and sex in the same row, differ significantly (*P* < 0.05). SEM: Standard error of the mean. Experimental diets: Control (TMR), UMS (20% untreated maize stover), AMS (20% ammoniated maize stover) and AMSVL (10% ammoniated maize stover: 10 *Vachellia* leaves)

## Discussion

The major challenges observed when ammoniated stover has been used in ruminant feeding systems are the feed intake depression and toxicity, which are related to several factors such as total daily ingestion of ammonia, dietary CP and proportion of the CP supplied as non-nitrogen protein. In order to prevent these adverse effects, inclusion level of 20% AMS was used in the current study as recommended in previous studies (Chelopo and Marume 2022; Dayani et al. 2011). The increase in feed intake in lambs fed the AMSVL diet can be attributed to the effect of *Vachellia* leaf meal could have improved rumen fermentation and nutrient availability as observed in other studies (Abdul et al. 2014). The improved intake in the AMSVL fed lambs could also be due to the diluent effect of the leaf meal on the high ADF and NDF that characterize maize stover in comparison with the normal fibre source in the total mixed ration as observed elsewhere (Undi et al. 2001). Although ADFI for lambs fed on AMSVL diet was slightly lower than that of the control diet, observation from the results showed that the inclusion of the leaf meal substantially increased the feeding value of the diet and the feed intake when compared to the AMS and UMS respectively. The differences in ADFI between lambs fed on UMS and AMS, shows that ammonia treatment also improved the quality of the stover and ultimately the utility of the treated maize stover as reported elsewhere (Huang et al. 2022; Mahesh and Mohini 2013; Souzza et al. 2010; Undi et al. 2001). This is because ammoniation exposes rumen degradable protein that is locked between the lignin bonds in untreated maize stover.

Although ADG and FCR were statistically not affected by diet, numerically, lambs fed on a diet containing AMS and AMSVL had marginally higher ADG and FCR as compared to the control and the UMS diets. The lower body weights obtained in lambs fed the UMS diet confirms the fact that untreated maize stover on its own is unable to release sufficient nutrients to meet the ruminant’s requirements even if it had similar CP to all dietary treatments, owing to the effect of the fibrous nature of untreated maize stover on supply parameters such as voluntary feed intake. This is consistent with reports from other studies which showed poor growth and productivity in ruminants fed only on maize stover-based diet (Barde et al. 2010; Ngongoni et al. 2009). Improvement in growth performance of lambs fed on combined inclusion of AMS and leaf meal was expected based on their nutritive value and a probable increase in digestibility of the diets. Metawi et al. (2017) also confirm improved performance in goats supplemented foliage leaves.

Maize stover is known to have lower digestibility, and as the digestibility of the diet decreases, inefficient fermentation occurs resulting in its energy being converted to CH_4_ gas (Metawi et al. 2017). Lambs fed the UMS diet had the highest CH_4_ gas emission compared to all other dietary treatments, with the control diet producing the least. The high CH_4_ production in lambs fed the UMS inclusion diet may be due to maize stover having a slow passage rate, prolonged residency time in the rumen, and inefficient fermentation while a comparative reduction in CH_4_ emission in AMSVL fed lambs could be due to the increase feeding value and quality of the diet with the inclusion the leaf meal. Inclusion of AMS alone decreased CH_4_ emission by 26%. However, the combination of AMS and *Vachellia* leaf meal inclusion led to a 36% decrease in CH_4_ emission compared to the UMS diet. Therefore, treatment of stover and inclusion of *A. erioloba* leaf meal in diets has the potential to improve feeding value and nutritive quality of diets, which consequently improves digestibility, whilst reducing CH_4_ emission. This is in line with findings by Zhang et al. (2019) who reported a gradual decrease in CH_4_ emission when feeding urea-treated rice straw on a small ruminant. *Vachellia* leaf meal inclusion in highly fibrous diets is known to have the ability to lower methanogenesis indirectly through inhibition of the growth of archaea methanogens (Tavendale et al. 2005). The effect of *Vachellia* leaf inclusion on CH_4_ emission reduction is also linked to the presence of some secondary plant metabolites (Benchaar et al. 2008; Metawi et al. 2017; Montoya-Flores et al. 2020).

Blood profiles provide a significant basis for the assessment of the health and nutritional status of animals. They also provide a good indication of the effects of different extraneous factors on health, immune response, and normal physiological functioning of the body (Beigh et al. 2018). The hematological values obtained in the current study were within the expected ranges (Beigh et al. 2018) for all diets. The observed reduction in RBC in AMSVL fed lambs is consistent with the decrease in the hematocrit value. Hematocrit values indicate the adequacy of the supply of healthy red blood cells, which, when deficient may result in anemia. *The Vachellia* species contain secondary plant compounds such as tannins. Intake of condensed tannins (CT) may precipitate proteins that inhibit the absorption of iron and reduce the bioavailability of iron (Rani et al. 2018) hence the observed results. In contrast Paswan et al. (2016) and Brown et al. (2018) reported no effect on RBC with the inclusion of *Vachellia* leaves. Lower basophil count may be due to the suppressive effect of CT contained in the *Vachellia* leaves. This was in agreement with Brown et al. (2018), Olafadehan (2011) and Solaiman et al. (2010) who reported a reduction in the WBC component when the animals were fed on CT rich diet.

Serum biochemical parameters are also bio-indicators of the nutritional and general health status of the animals. The results of the current study show an increase in albumin (ALB), amylase (AMYL), and total bilirubin (TBIL) values with the inclusion of *Vachellia* leaves in a diet. The observed increase in ALB indicates an increased amount of available metabolic protein to the animal contributed by the *Vachellia* leaf meal as observed in other studies (Zhao et al. 2019). The observed increase in ALB and AMYL concentrations in the AMSVL fed lambs compared to other diets may reflect better protein and energy digestion and utilization for that diet. TBIL was also significantly higher in lambs fed on AMSVL inclusion diet, however, their values remained within the reference range of 0.88 to 8.84 µmol/L (Borjesson et al. 2000). The observed high levels of bilirubin in lambs fed the AMSVL diet could be associated with the presence of polyphenolic compounds including flavonoids and tannins in the Vachellia leaf meal which is normally causes the elevation of the bilirubin concentrations (Gupta et al. 2005). The trend of slightly increase total bilirubin with inclusion of leaf meal may indicate some effects of leaf meal on the immune system which is related to tannin concertation of Vachellia species. However, the current study revealed no damage to liver and kidney function parameters. This may be due to acceptable inclusion or dosage of the leaf meal in lamb diet. Serum AMYL activity was significantly higher in lamb fed on a diet with combined inclusion of AMS and *AL*. This may be attributed to higher particles involved in fibre digestion of the stover and nutrient availability in *Vachellia* leaves. An increase in AMYL activity in lamb fed on combined inclusion of AMS and *AL* was expected as the AMYLY activity depends mostly on improved fibre digestion in the rumen (Raghuvansi et al. 2007).

Calcium (Ca), Alkaline transferase (ALT), cholesterol (CHOL), creatinine (CREA), globulin (GLOB), glucose, lipase (LIPA), phosphorus (PHOS) and total protein (TP) were not affected in all the dietary treatments. Solaiman et al. (2010) reported no effect Ca, ALT, AMYL, CHOL, CREA, GLOB, GLU, LIPA, PHOS and TP of goats consuming CT containing forage. In contrast to the results of the current study other studies confirm a decrease in TP with the inclusion/supplementation of *Vachellia sp* leaves (Zhao et al. 2019). Sallam et al. (2017) also observed reduced TP with partial substitution of clover hay by *Vachellia sp*. The current study shows no significant effect of diet on TP across all the dietary treatments, however, numerically lambs fed on a diet containing *Vachellia* leaves (AMSVL) had higher serum TP and more comparable to control diet-fed lambs, this could be enhanced by the interaction of *Vachellia* leaves and ammoniated stover as observed in as study by Sallam et al. (2017).

Surprisingly, with no effect on the serum TP, the serum urea nitrogen was significantly high in lambs fed on AMS with values that were above the normal range of 2.8–7.1 mol/L as outlined in the Merck and Manual (2012) for healthy lambs. The possible reason for an increase in serum urea could be as a result of rumen undegradable protein supplied through ammoniation. The increase in serum urea due to ammoniation agrees with the findings reported by Kraidees (2005). Blood urea is an indirect indicator of the protein composition of feed associated with greater ruminal degradation of protein with a concurrent increase in ammonia production (Broderick and Clayton 1997; Soul et al. 2019). As expected, the serum urea level was reduced with the inclusion of *Vachellia* leaves. Sallam et al. (2017) reported a reduction in blood urea in goats fed *Vachellia saligna* leaves. Waghorn et al. (1994) also concluded that *Vachellia* supplementation has the potential to lower serum urea in sheep. Both hematological and serum biochemical parameters observed were all within normal ranges for healthy growing lambs (Merck manual 2012), except for serum urea in lambs fed on the AMS diet. This implied that the test diets were able to supply an adequate amount of nutrients needed to maintain normal lamb’s blood metabolites except for AMS based diet on serum urea.

## Conclusion

Inclusion of *Vachellia erioloba* leaf meal in AMS based diet significantly increased feed intake and reduced CH_4_ emissions, nutritional and health status of the lambs, as it had the potential to increase the serum albumin, which is the main component indicator of the nutritional and health status of the animal. The desirable blood metabolite levels observed in the current study indicate the potential of the combined effect of AMSVL inclusion in improving the general health status of lambs. Use of ammoniated maize stover with *Vachellia* leaves could be implemented in lamb finisher feeding without detrimental effects on growth performance and health status of the animals but rather improve growth performance and health status of the lambs.

## Data Availability

The data sets used in the current study are available from the corresponding author on request.
